# Emerging Treatments for Persistent Corneal Epithelial Defects

**DOI:** 10.3390/vision9020026

**Published:** 2025-04-01

**Authors:** Jeonghyun (Esther) Kwon, Christie Kang, Amirhossein Moghtader, Sumaiya Shahjahan, Zahra Bibak Bejandi, Ahmad Alzein, Ali R. Djalilian

**Affiliations:** 1 Department of Ophthalmology and Visual Sciences, University of Illinois at Chicago, Chicago, IL 60612, USA; kwon16@uic.edu (J.K.); moghtada@uic.edu (A.M.); sshahj2@uic.edu (S.S.); zhrbibak@uic.edu (Z.B.B.); aalzei2@uic.edu (A.A.); 2 Department of Physiology and Biophysics, University of Illinois at Chicago, Chicago, IL 60612, USA; ckang30@uic.edu

**Keywords:** Persistent Corneal Epithelial Defect, Persistent Epithelial Defect, Neurotrophic Keratitis, Corneal Neurotization, Limbal Stem Cell Deficiency, Limbal Cell Reconstruction, Ocular Surface, Cornea

## Abstract

Persistent corneal epithelial defects (PCEDs) are a challenging ocular condition characterized by the failure of complete corneal epithelial healing after an insult or injury, even after 14 days of standard care. There is a lack of therapeutics that target this condition and encourage re-epithelialization of the corneal surface in a timely and efficient manner. This review aims to provide an overview of current standards of management for PCEDs, highlighting novel, emerging treatments in this field. While many of the current non-surgical treatments aim to provide lubrication and mechanical support, novel non-surgical approaches are undergoing development to harness the proliferative and healing properties of human mesenchymal stem cells, platelets, lufepirsen, hyaluronic acid, thymosin ß4, *p*-derived peptide, and insulin-like growth factor for the treatment of PCEDs. Novel surgical treatments focus on corneal neurotization and limbal cell reconstruction using novel scaffold materials and cell-sources. This review provides insights into future PCED treatments that build upon current management guidelines.

## 1. Introduction

PCEDs are defined as the failure of the re-epithelialization of the cornea within 14 days of standard care after an insult or injury [1,2,3]. The annual incidence of PCEDs in the United States is estimated to be less than 100,000 cases [4]. The condition arises from a diverse array of etiologies, including limbal stem cell deficiency (LSCD), autoimmune diseases, systematic inflammatory diseases, adhesion dysfunction, recurrent corneal damage from repeated mechanical trauma, and neurotrophic damage [1,5]. Neurotrophic changes to the trigeminal nerve could be secondary to infectious causes (HSV, HZV, acanthamoeba), systemic diseases (diabetes mellitus), iatrogenic etiologies (ocular surgeries, medication induced), neurologic reasons (trigeminal nerve palsy), and congenital diseases (congenital corneal hypoesthesia) [6]. Although the exact percentage of different PCED etiologies is unknown, some of the prevalences of the following etiologies have been reported. A study reported the incidence of PCEDs or corneal ulcers as 25.4% in patients who received corneal transplantation [7]. Of 250,000 diabetic vitrectomy surgeries per year in the US, 2480–5257 had iatrogenic PCEDs [4,8]. In the US, 20.7 people for every 100,000 people develop PCED secondary to ocular herpes simplex annually [4], and 72% were reported to have corneal–epithelial involvement [9]. Of the 40,000–60,000 cases of herpes zoster ophthalmicus per year, corneal involvement comprises 1600–4800 cases [4]. However, the incidence of PCEDs of the corneal–epithelial involvement is unclear.

### 1.1. Pathophysiology of PCED

The corneal wound healing process includes a series of events: “migration, proliferation, adhesion and differentiation with cell layer stratification” [10]. Some keratocytes in the stroma go through the initial apoptosis. However, some quiescent keratocytes undergo activation, migrate to the defect [11], and secrete a variety of growth factors essential for effective wound healing [1,5,10,12]. The integrity of the basement membrane is critical for maintaining the fibroblast population. When the basement membrane is damaged or disrupted, corneal epithelial cells secrete TGFβ2 [11], which induces the transformation of fibroblasts to myofibroblasts [11,13]. The regenerated epithelium adheres to the uninjured stroma via hemidesmosomes in a week and up to eight weeks if the stroma is also damaged [5]. When any steps of the described wound healing processes are impeded, the corneal epithelial defect can persist for longer than two weeks and is considered a PCED. PCEDs are primarily caused by abnormalities in epithelial adhesion, limbal stem cell deficiency, inflammation, or neurotrophic pathways.

The epithelium cannot attach to the basement membrane when hemidesmosomes between the two layers are altered. Recurrent corneal erosions, epithelial basement membrane dystrophies, toxic preservatives in eye drop, or corneal degenerative diseases can also interrupt epithelial adhesion [5].

Another common cause of PCEDs is LSCD. Corneal epithelial stem cells promote epithelial regeneration after corneal injury [14]. They reside at the limbus [15], respond to central corneal wounds [15], and migrate centripetally [16].

When inflammatory cascades are activated, the release of cytokines, such as interleukin-1β, tumor necrosis factor α, interleukin-6, interleukin-8; matrix metalloproteinases (MMP); and chemotactic factors is increased for tissue remodeling [2,5]. When the homeostasis of the pro-inflammatory is not maintained, inflammation can be further exaggerated by activated immune cells [2,17]. Consequently, epithelial cells are damaged, compromising the epithelium barrier and hindering regeneration [2]. Patients with systemic inflammatory conditions or autoimmune conditions, such as Stevens–Johnson syndrome, graft-versus-host disease, rosacea, rheumatoid arthritis, Sjögren’s syndrome, etc., are at risk of persistent corneal inflammation [1,5].

PCEDs, as a sequala to neurotrophic damage of the trigeminal nerve, comprise newly categorized neurotrophic keratopathy of stage 3 and above [6]. Recently, the Neurotrophic Keratopathy Study Group has agreed on three main contributing factors to the pathophysiology of NK: altered corneal sensation, decreased trophic factors, and altered lacrimal function unit [6]. The trigeminal nerve, a peripheral nerve, has regenerative capacity [18]. However, when this regenerative ability is compromised, neurotrophic changes can occur. Consequently, the blink reflex, trophic factor release, and tear production are altered. These changes lead to the breakdown of the corneal epithelium, decreased clearance of toxic substances or inflammatory cytokines on the ocular surface, and decreased wound healing from decreased neurotrophic factor releases [6].

### 1.2. Overview of Diagnostics and Management of PCED

The diagnosis of PCEDs is established clinically. The size of the epithelial defect can be measured under a slit lamp with fluorescein staining, while the depth of the defect can be assessed using anterior segment optical coherence tomography [19]. Slit lamp evaluation helps to identify the presence of infection and the severity of inflammation. Various etiologies of PCEDs should be evaluated. Any abnormalities of the eyelids, blinking, and tear film should be evaluated as all of these can contribute to PCEDs. PCEDs, in the context of NK, can be assessed by measuring the corneal sensitivity. Certain labs can be ordered to evaluate systematic comorbidities such as HbA1C for diabetes, serum retinol-binding protein for vitamin A deficiency, and various antibody panels (anticyclic citrullinated peptide, antinuclear antibody, anti-double-stranded DNA antibody, anti-Ro, and anti-La) for autoimmune diseases [1].

The current non-surgical standard of care for PCEDs includes aggressive lubrication with preservative-free artificial tears, punctal plugs to mechanically maintain lubrication, treatment of underlying conditions, discontinuation of any offending agents, contact lenses, scleral contact lens to protect against mechanical insult, oral tetracyclines to dampen matrix metalloproteinase (MMP) activity, and administration of prophylactic antibiotics and steroids [1,5,20,21,22,23]. Refractory cases to the standard of care can be treated with newer medical management such as autologous serum or platelet-rich plasma tears [24]. PCEDs can be surgically managed with tarsorrhaphy, amniotic membrane transplant (AMT), corneal epithelial stem cell transplantation, and conjunctival flap [2,5,25,26,27,28,29,30].

### 1.3. Purpose

Timely intervention is imperative to prevent complications associated with unhealed PCEDs, such as increased exposure to infections, corneal scarring, opacification, melting, perforation, and ultimately vision loss [1,31,32]. Research indicates that a longer PCED duration may be a risk factor underlying complications but also a prognostic indicator for requiring extended treatments [31,32]. This review delves into current management strategies and highlights emerging non-surgical and surgical treatment options. Relevant articles were searched with terms such as “persistent epithelial defect”, “persistent corneal epithelial defect”, “neurotropic keratitis”, “neurotropic keratopathy”, “clinical trials”, “emerging treatments”, “surgical managements,” “limbal stem cell deficiency,” “scaffolds”, “cell therapy”, “current surgical management”, and “emerging surgical management” on MEDLINE, PubMed, Google Scholar, ClinicalTrials.gov, International Clinical Trials Registry Platform, and EU Clinical Trials Register.

## 2. Current PCED Management

### 2.1. Current Non-Surgical Management

Managing PCEDs involves optimizing the ocular surface to promote effective healing (Table 1). The primary goal of management is to create an environment that supports cellular repair and to minimize the risk of further epithelial breakdown. To effectively treat PCEDs, both the underlying cause and symptoms must be considered. Following identification of the underlying cause, a stepwise system of treatment should be employed, beginning with conservative measures and progressing to more aggressive treatment options if refractory.

- Treat the Underlying Cause: The initial step in managing PCEDs is to determine the underlying cause. Addressing the underlying cause is necessary for maximizing the efficacy of subsequent local therapies [24]. For example, PCEDs secondary to autoimmune causes may require treatments that focus on suppressing the inflammatory response, and PCEDs secondary to LSCD may need limbal cell transplant. It is also important to survey for iatrogenic causes. Ophthalmic eye drops that contain preservatives like benzalkonium chloride can damage the integrity of the corneal epithelium [43]. Antibiotics, antivirals, and medications commonly prescribed for glaucoma management are associated with corneal toxicity [21,24].
- Aggressive Lubrication: The first line of treatment for PCEDs is aggressive lubrication of the cornea with preservative free artificial tears and ophthalmic ointments [21]. Lubrication supports the healing process by promoting epithelial cell migration, reducing mechanical trauma, and enhancing the stability of the tear film [33].
- Punctal Plugs: Punctal plugs are another therapeutic method employed to enhance corneal lubrication and promote healing. By obstructing tear drainage, they increase the retention time of natural tears on the ocular surface, thereby enhancing hydration and supporting the healing process [21]. Since punctal plugs create a reservoir of lubrication, they must be used cautiously when the patient is on the cytotoxic agents in commonly prescribed drops such as antibiotics, antivirals, and glaucoma medications. With the drainage system blocked, the ocular surface is exposed to an increased level of toxins due to prolonged retention. This blockage is particularly concerning in patients chronic ocular surface diseases as their reduced corneal sensitivity may delay the detection of inflammation, potentially exacerbating ocular surface damage [21].
- Contact Lens: A bandage or scleral contact lens is also indicated to serve as a protective barrier between the PCED and the environment [20,34]. By providing a physical barrier, lenses allow the epithelial defect to heal without reoffending trauma and irritation. Scleral contact lenses also provide a reservoir of lubrication underneath the lens, further optimizing the corneal surface for healing [2].
- Pressure Patching: Pressure patching is an alternative approach to providing a protective barrier to the cornea. Placing two eye pads over the eye and securing them with tape creates a physical barrier, while the added pressure helps prevent lid movement, stabilizes the cornea, and supports epithelial regeneration [44]. However, pressure patching may confer a negative effect by limiting oxygen availability, potentially hindering its regeneration. As a result, its use remains controversial in clinical practice [44,45].
- Oral Tetracyclines: Oral tetracyclines taken systemically can effectively treat PCEDs. The anticollagenolytic activities of tetracyclines are due to the resulting inhibition of MMPs involved in the inflammatory process associated with PCEDs [5]. By suppressing MMP activity, tetracyclines can promote healing when there is an inflammatory component to the PCED [35]. However, prolonged usage of oral tetracyclines should be monitored as it has been shown to significantly decrease gut microbiota diversity without recovery post antibiotic withdrawal [36]. Furthermore, broad usage of this antibiotic leads to an increase in the bacterial acquisition of the tetracycline resistance gene tetW among certain strains of the bacterium [36].
- Autologous Serum Drops (ASDs): ASDs are created from autologous blood serum. ASDs contains many of the growth factors and nutrients, such as vitamin A and epidermal growth factor, that are present in a healthy tear film yet deficient in the tears of PCED patients [3]. In addition, fibronectin and anti-proteases in ASD are some of the factors in serum believed to promote corneal healing [37]. A case study of a 28-year-old patient reported the effective and complete healing of a two-month-old corneal epithelial defect after 48 h of ASD application six times daily [46]. Platelet-derived therapeutics are discussed under “3. Emerging Non-surgical Management”.
- Other Biologics: The REPARO phase I and phase II studies have demonstrated the safety and efficacy of recombinant human nerve growth factor (rh-NGF) eye drops in treating moderate to severe neurotrophic keratitis (NK), a condition where impaired corneal innervation leads to epithelial defects [47,48]. Subsequently, cenegermin (an rh-NGF) received regulatory approval for NK treatment from agencies such as the European Commission, the United States Food and Drug Administration, and other authorities worldwide [38]. Further investigations have supported the role of rh-NGF in managing PCEDs associated with NK. Ref. [39] reported complete healing in all eight patients with moderate NK and PCEDs after an 8-week treatment of six daily drops of cenegermin, with three patients achieving healed PCEDs within 4 weeks [39]. Similarly, ref. [38] demonstrated that cenegermin achieved statistically significant greater reductions in corneal lesion size compared to vehicle-treated controls over an 8-week treatment period [38]. Additionally, cenegermin has shown efficacy when used in conjunction with bandage contact lens. Cheung et al. [6] reported complete resolution of the PCED in 6 out of 10 NK patients and a notable reduction in defect size in three additional eyes at the end of the treatment period [40]. These findings highlight the therapeutic role of cenegermin in addressing PCEDs when neurotrophic dysfunction is a driving factor. Insulin is another biologic that is used for PCED healing. A prospective study reported approximately a 70% reduction in defect size among 23 patients with PCEDs [41]. Similarly, a prospective case series on 10 patients with PCEDs treated with insulin eye drops showed complete re-epithelialization in 82% of the patients [42].

### 2.2. Current Surgical Management

Surgical management (Table 2) is typically reserved for severe cases that are resistant to other topical treatments. The decision on the priority of the surgical technique totally depends on the underlying disease causing the PCED. Depending on the situation, one or a combination of surgical techniques can be most effective. Surgical management choices will be dependent on the individual patient presentation and medical and surgical history.

- Amniotic Membrane Transplant (AMT): The amniotic membrane can promote epithelialization and exhibits anti-inflammatory and anti-scarring properties, making it an effective dressing for various conditions, such as PCEDs with corneal ulceration, acute chemical burns, bullous keratopathy, and LSCD [56]. In an animal study, rabbits with pseudomonas keratitis and PCEDs were treated with AMT, ciprofloxacin, or both [57]. Corneal perforation was present in 85.6% of rabbits in the control group but absent in all treatment groups [57]. The combination of ciprofloxacin and AMT demonstrated the best results in reducing infiltrate size [57]. Additionally, eleven eyes of patients with PCEDs (mean defect area: 13.2 ± 11.3 mm^2^) underwent AMT, with complete resolution achieved in eight eyes after the initial AMT and in three additional eyes following a second transplantation [58].
- Tarsorrhaphy: Tarsorrhaphy is a temporary or permanent procedure to partially or completely close the eyelid for corneal healing. It provides protection against trauma and various exposures. This can be achieved through sutures, botulinum toxin (i.e., to paralyze the upper lid levator muscle), cyanoacrylate glue, or with the addition of a weight (e.g., gold) to the eyelid [59]. In a study of thirty-four eyes with PCEDs of various etiologies, tape splint tarsorrhaphy (TST) achieved complete epithelial defect healing in 85.3% of cases after an average of 22.5 ± 24.3 days, without requiring additional treatment. Best-corrected visual acuity (BCVA) improved from 1.11 ± 0.41 to 0.83 ± 0.70 (*p* = 0.02). TST is a cost effective and efficient treatment for managing PCEDs [49].
- Conjunctival Flap (CF) Surgery: Vascularization of the CF provides the necessary nutrients for corneal wound healing [2], dampens inflammation, and alleviates pain [50]. However, the fine balance required to apply appropriate tension in suturing can add complexity to conventional suturing [50]. CF with fibrin glue instead of conventional sutures showed compatible efficacy results without any conjunctival retraction. All seven patients achieved a stable ocular surface [50]. The use of fibrin glue shortens operation duration and fastens recovery [50]. However, CF is reserved for severe PCEDs because of increased risk of LSCD development, corneal conjunctivalization, neovascularization, and opacification [2,60].
- Limbal Stem Cell Transplantation: LSCD is a condition that often leads to PCEDs due to an insufficient number of limbal stem cells in corneal surface repair. Management depends on whether the condition is unilateral or bilateral. For unilateral conditions, a block of the conjunctival–limbal autograft (CLAU) from the intact fellow eye is transplanted onto the affected eye [51]. Another technique to transplant the limbal cells is cultivated limbal epithelial transplantation (CLET), in which cells are expanded ex vivo before transplantation. A study with participants with bilateral LSCD who underwent CLET showed 71.4% of successful construction after four years [52]. Sangwan [53] et al. proposed simple limbal epithelial transplantation (SLET) by combining the benefits of CLAU and CLET. In this technique, an amniotic membrane is transplanted onto the denuded recipient’s ocular surface with the basement membrane side facing up. Subsequently, 8 to 10 small pieces of limbal tissue from a healthy donor are placed and fixed on the amniotic membrane with the epithelial side facing up [52]. Finally, a bandage contact lens is applied to secure the graft [53,61]. The efficacy of SLET was proved in a study in which 95 of 125 eyes (76%) had successful limbal transplant and maintained corneal stability at final visit, which ranged from 1 to 4 years [62].
- Keratoplasty: Keratoplasty choice depends on the underlying disease causing the PCED. In both LSCD and neurotrophic conditions, the loss of both limbal stem cells and neuronal support means that simply replacing the cornea does not provide a long-term solution; it only offers short-term improvement [54]. Keratoplasty can be performed either simultaneously with or after limbal reconstruction. A similar challenge arises in neurotrophic conditions, where corneal transplantation alone is insufficient for sustained healing. Neuronal replacement and reconstruction are needed prior to keratoplasty. It serves as a complementary procedure following limbal and neuronal reconstruction or when stromal melting occurs in severe corneal wounds [54]. Mini-conjunctival limbal autograft (CLAU) combined with deep anterior lamellar keratoplasty (DALK) resulted in complete epithelialization and a stable long-term outcome in a patient with total LSCD [63]. Penetrating keratoplasty combined with autologous simple limbal epithelial transplantation (SLET) in severe stromal and surface scarring resulted in a transparent cornea and a visual acuity of 20/100 [60]. In 14 patients who underwent COMET, PKP was performed 7.6 ± 1.3 months later. Over a follow-up period of 28.2 ± 8 months, no recurrence of corneal defects was observed [51]. Keratolimbal allograft (KLAL) combined with central lamellar keratoplasty (CLK) was performed in 13 patients with chronic and severe mustard gas keratopathy (MGK), with a reported success rate of 92.3% over a follow-up period of 87.6 ± 49.8 months [64].

## 3. Emerging Non-Surgical Management

There are a few emerging medical treatments that are on the rise (Table 3).

### 3.1. Platelet-Derived Therapeutics

Platelet-rich plasma (PRP) is an emerging treatment modality in regenerative medicine [83]. PRP, which can be further formulated as a platelet gel, has been recognized for its role in tissue repair [65]. To produce PRP, the patient’s blood is anticoagulated with animal-derived thrombin and centrifuged. The PRP layer between the platelet-poor plasma layer and the white/red blood cells layer is aspirated [65,68,70]. PRP is rich in platelet-derived growth factor (PDGF), which is known to be the first responder in cell regeneration and therapeutic angiogenesis [68]. Studies have shown that PRP promotes wound healing in corneal epithelial injury [84] and PCEDs [65,66]. An ongoing clinical trial aims to build upon this potential by studying combination therapies to determine whether supplementing PRP drops with a bandage contact lens, an eye patch with lubricant ointment, or preservative-free lubricant alone produces the quickest healing times for PCEDs [67].

PRP can be formulated not only into topical eyedrops but also clots [69]. Solid-activated PRP, the “clot”, was traditionally applied with a sutured amniotic membrane. Recently, solid-activated PRP is being used in conjunction with a soft contact lens, which is more accessible and cost-effective [85]. This treatment can be effective especially among patients who have contraindications for amniotic membrane usage due to underlying systemic conditions, infections, or ineligibility to be a blood donor [86].

Another platelet-derived treatment modality is platelet-rich in growth factors (PRGF), in which calcium chloride is used as a coagulant instead of animal-derived thrombin [87]. The PRGF production technique avoids collection of the buffy coat and white blood cells [70]. PRGF has a high concentration of growth factors, including not only PDGF but also epithelial growth factor, vascular endothelial growth factor, hepatocyte growth factor, fibroblast growth factor, and nerve growth factor, which aid in the ability of PRGF to improve ocular surface quality [88,89]. In a study involving 20 PCED cases, the application of PRGF eye drops led to complete resolution of the PCED in 17 patients within an average of 10.9 weeks [89].

Additionally, platelet lysate solutions are undergoing development and trials. The process of thermal shocking diluted PRP lyses platelets, which can then be collected as a potential therapeutic solution [70]. In an animal model, platelet lysate was delivered via an ocular wound chamber and was found to be effective in the re-epithelialization of the cornea [71]. In a pilot clinical trial involving 10 patients with PCEDs, 7 patients had a complete resolution of their PCEDs with autologous platelet lysate [72].

### 3.2. Ocular Bandage Gel (KIO-201)

Hyaluronic acid is a glycosaminoglycan that plays a vital role in the proper function of the cornea. Due to its viscoelastic properties, hyaluronic acid contributes to the structure and plasticity of the cornea [90,91]. Kiora Pharmaceuticals is developing an ocular bandage gel that uses a more stable, cross-linked form of hyaluronic acid called KIO-201 for PCED treatment. In 2023, the completion of an interventional phase 2 study of KIO-201 revealed that five of the eight participants showed an appreciable reduction in the lesion area by the end of the four-week treatment period [73,74].

### 3.3. Lufeprisen Ophthalmic Gel (AMB-01-006 or NEXPEDE-1)

Amber Ophthalmics is advancing Nexagon (lufepirsen ophthalmic gel), which inhibits the formation of connexin43 gap junction hemichannels. This inhibition has been shown to promote the healing of corneal epithelial defects and vascular recovery [77]. Amber Ophthalmics is currently recruiting for phase 2 and phase 2/3 clinical trials to evaluate its safety and efficacy in PCED management, measuring the proportion of participants that achieve corneal re-epithelialization and maintain re-epithelialization within 28 days [75,76,78].

### 3.4. The 0.1% RGN-259 Eye Drops

A corneal nerve disorder or damage in NK leads to PCEDs. Stage 2 NK is defined by the presence of a PCED, while Stage 3 NK is characterized by the progression to corneal ulceration, stromal melting, or corneal perforation [92]. Chronic wounds are associated with the overproduction of inflammatory cytokines and chemokines, preventing proper wound healing. Thymosin ß4, an active form of thymosin fraction 5, promotes wound healing by decreasing apoptosis, prevents scarring by decreasing myofibroblasts, strengthens cell-to-cell adhesion by increasing laminin-5 synthesis, and provides oxygen by increasing angiogenesis [93]. In a phase 3 clinical trial, thymosin ß4 was formulated into 0.1% RGN-259 eye drops and administered to 10 patients with PCEDs and stage 2 or 3 NK. Using a five-times-daily dosing schedule, the complete resolution of the PCED occurred in six patients within 28 days [79].

### 3.5. FGLM-NH2 + SSSR Eye Drops

FGLM-NH2, which is a substance *p*-derived peptide, and SSSR, which is a derivative from the C-domain of insulin-like growth factor-1, promote epithelial regeneration [80,81]. A combination of FGLM-NH2 and SSSR was applied four times a day for 90 days to 11 patients with NK in the context of leprosy. Of the five patients with PCEDs, all five patients saw a size reduction in their defects, and four patients experienced complete resolution of their PCEDs within 3–14 days of starting the eyedrops [82].

## 4. Emerging Surgical Management

New studies on surgical management of PCED, including corneal neurotization and cell-based therapies, are under investigation (Table 4).

### 4.1. Corneal Neurotization

Corneal nerves offer a protective and reparative ecosystem that is essential for optimal corneal healing. Corneal neurotization is a procedure that involves the transplantation of a well-functioning sensory nerve to the damaged cornea [112]. Both direct and indirect corneal neurotization techniques aim to restore corneal innervation. In direct corneal neurotization (DCN), supraorbital or supratrochlear nerves on one side of face are transferred to the other side of face under the nasal bridge [94]. Although DCN can sensitize the cornea, DCN is not applicable to patients with bilateral palsies and leaves extensive scars from bi-coronal incisions [113]. Elbaz et al. developed indirect corneal neurotization, in which the sural nerve graft is used to interconnect the ends of the supraorbital or supratrochlear nerve to the contralateral ocular surface through sub-brow incisions [113].

Nerve ends can be connected in three different ways: end-to-end (ETE), side-to-side (STS), or end-to-side (ETS) [112,114,115,116]. The donor and recipient nerves need to be of a similar size for the ETE approach [117]. However, if there is a significant difference in nerve size or function, the ETS approach may be a more suitable alternative [112]. In a multi-center review, 16 patients with NK who underwent DCN were followed for an average of 31.3 months post-procedure. On average, corneal sensitivity improved from 3.6 mm to 25.3 mm. Additionally, 9 patients demonstrated improved visual acuity, and 11 patients demonstrated an increase in corneal stability [118].

### 4.2. Cell Therapy-Based Approaches Under Investigation

PCEDs secondary to LSCD require the replenishment of limbal stem cells to promote stem cell migration to the cornea and enhance corneal healing. PCEDs in the context of LSCD may need surgical management [14]. For limbal reconstruction, two essential factors must be considered: an appropriate cell source and the scaffold. For cell replacement, a surface is needed to seed and hold the cells [119]. The amniotic membrane is a well-established biomimetic scaffold used for corneal reconstruction. With its anti-inflammatory and regenerative properties, the amniotic membrane has been used most frequently for cell seeding. Despite its favorable results in culturing various cell types [120,121,122], the amniotic membrane has fallbacks of donor-dependent variations [123] and increased risks of infection and rejection [97]. The following are emerging biomimetic and artificial scaffolds under investigation.

- Plywood-like Matrices: Human corneal epithelial cells were cultured on plywood-like matrices for 14 days and were compared with the cells cultured on amniotic membrane [95]. The results proved the compatibility of plywood-like matrices with amniotic membrane with a greater cell amplification [95].
- Compressed Collagen Gels: Compressed collagen gels are better suited for corneal limbal epithelial cell expansion than their traditional collagen gel counterparts, which are uncompressed. The compression of these gels allows for stronger attachments between the cells themselves, as well as between the cells and their environment, providing a supportive construct for more morphologically uniform cell growth [96].
- Hyaluronan (HA): HA stimulates corneal epithelial migration [98]. Preclinical studies have used HA as scaffolds to expand corneal epithelial stem cells ex vivo [97] and to culture human oral mucosal epithelial cells (OMEC) [124].
- Chitosan: Chitosan is a bioresorbable polysaccharide, which is made of chitin, a natural polymer [99]. This is another novel material that may be used as a scaffold for limbal reconstruction. An animal study showed that the alginate–chitosan hydrogel can be successfully used as a scaffold for corneal reconstruction in situ [100].

The source of the cells plays a critical role in corneal healing. In unilateral LSCD, the most favorable option is the use of contralateral autologous limbal stem cells [125]. For bilateral LSCD, allograft limbal stem cells or alternative cell types that have demonstrated potential for differentiation, as listed below, can be utilized for ocular surface healing [126,127,128].

- Simple Oral Mucosal Epithelial Transplantation (SOMET): The oral mucosal epithelium is rich in stem cells and can serve as an autologous graft for corneal epithelial restoration [101]. At the 55-month follow-up, 95% of the 17 patients who underwent autologous cultivated oral mucosal epithelial transplantation (COMET) reported improvements in BCVA and corneal scar healing [102]. A newer, simplified approach known as SOMET has since been introduced [103]. Initially performed in a rabbit model, this technique involved securing small rabbit buccal grafts on the cornea with a contact lens. After two weeks, the treatment group had complete resolution of LSCD with signs of ocular surface healing such as reduced neovascularization and decreased fluorescein staining [104]. Additionally, a case report shows the potential efficacy of SOMET in a patient with chronic bilateral ocular sequelae of Stevens–Johnson syndrome. Three weeks after SOMET, the patient’s ocular surface was fully re-epithelialized [103].

MSC is a multipotent cell that can be differentiated into multiple cell types, including osteoblasts, adipocytes, and chondrocytes [129]. MSCs can be acquired from bone marrow, adipose tissue, the umbilical cord, the placenta, and menstrual blood [130]. A systematic review on MSC clinical trials reported that MSCs in research were most frequently derived from bone marrow [105]. MSCs have long been of interest for their ability to secrete anti-inflammatory and antiangiogenic factors [131,132,133,134,135]. In this segment, three different MSC interventions are reviewed: subconjunctival injection of MSCs, MSC transplantation, and MSC secretomes treatment.

**Figure 1:**
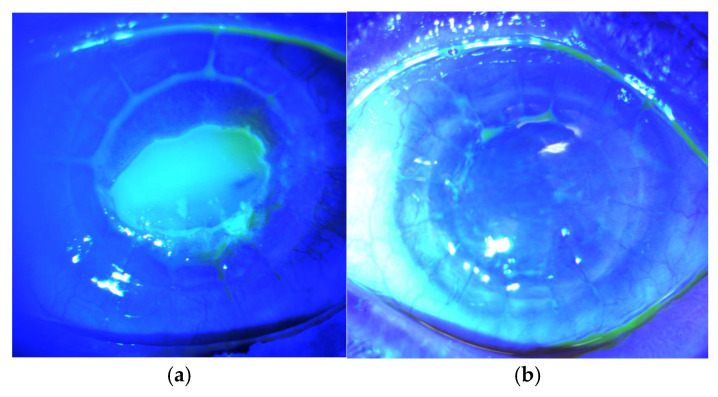
Phase I study—safety of locally delivered allogeneic mesenchymal stem cells for promoting corneal repair: (**a**) PCED size at baseline was 5.7 × 4.4 mm^2^; (**b**) complete resolution of PCEDs on Day 90.

- MSC Subconjunctival Injection: After establishing the safety of MSCs in animal model corneas [129], a single center, dose-escalation phase I clinical trial was conducted to test the safety of MSCs in patients with PCEDs [136]. The safety of locally delivered MSC via subconjunctival injection was proven without any occurrence of treatment-related toxicity [106]. Some efficacy was demonstrated (Figure 1), with reduced PCED size in five out of eight participants [106,107]. Its efficacy in improving the corneal epithelial barrier is currently under investigation in a multi-center, randomized, double-masked phase II study that started in 2024 [137].

- MSC Transplantation: A proof-of-concept clinical trial showed that allogeneic bone marrow-derived MSCT had comparable efficacy to cultivated limbal epithelial transplantation (CLET), with MSCT achieving an 85.7% and CLET achieving an 77.8% success rate at 12 months. Both approaches were deemed effective, safe, and free of adverse effects [108]. In a phase 2a clinical trial, 400,000 adipose tissue-derived adult MSCs were injected into the limboconjunctival quadrant in eight patients and covered by amniotic membrane. Over the 86.5 months follow-up, epithelial defects were resolved in all patients [109]. Other potential cell sources for corneal healing and limbal generation include dental pulp stem cells [127], hair follicle bulge-derived stem cells [128], and epidermal adult stem cells [126]; however, more studies are needed to assess their applicability.
- MSC Secretomes: Secretome is defined as “all factors actively or passively released from cells and contains among others soluble proteins (e.g., cytokines, chemokines and growth factors), lipids, free nucleic acids and extracellular vesicles” [138] (p.1). Interestingly, research showed that MSCs’ regenerative and immune-modulatory capabilities are due to paracrine signaling rather than the effects of direct cell transplantation [134,139,140,141]. As a result, akin to MSCs, MSC secretomes and the cultured media have antifibrotic [142], anti-inflammatory [134,143,144], antiangiogenic [114], and regenerative [133,143,145,146] effects. In fact, secretomes have a higher safety profile with low risk of infection, less time needed for expansion, and lower cost in production [147]. Our unmasked, single-center phase 1 clinical trial [110] proved the safety of topical MSC secretomes in low- (two times a day) and medium- (four times a day) dose cohorts. However, in the high-dose group (six times a day), there were two incidences of dose-limiting toxicity. Our preliminary data analysis showed that out of nine participants, seven patients demonstrated improved visual acuity, five patients had an increased corneal sensation, and six patients had fewer self-reported symptoms on the Ocular Surface Disease Index. Kala Pharmaceuticals have also formulated the MSC secretome into a topical ophthalmic solution, and they have named it “KPI-012”. Their phase 1b clinical trial also demonstrated encouraging results. Six out of eight patients showed complete healing of their PCEDs [148]. In early 2023, KPI-012 entered the phase 2 clinical trial phase and received FDA fast track designation, underscoring its potential as a transformative therapy for PCEDs [111]. This randomized, prospective clinical trial is still ongoing, with 47 study sites over the course of eight weeks [119,149].

## 5. Conclusions

Management of PCEDs can be challenging, especially due to a lack of response to standard care [24]. Delayed treatment of PCEDs increases the likelihood of corneal complications and, ultimately, can lead to vision loss. Researchers propose that patients at risk of recurrent PCEDs can be treated prophylactically and more aggressively in the early stages [24,31,32]. An emphasis on earlier treatments may result in a reduced number of follow-up visits and, consequently, less healthcare expenditure [24]. While the potential prophylactic use of the emerging treatments discussed remains uncertain, promising results from ongoing trials support their viability as treatment options for PCED.

## Figures and Tables

**Table 1:** Brief descriptions of current non-surgical managements.

Treatment	Descriptions
Treat the Underlying Cause	Address the primary cause like autoimmune disease or LSCD to optimize healing
Aggressive Lubrication	Preservative-free artificial tears and ointments that promote epithelial cell migration and improve the tear film stability [33]
Punctal Plugs	Block tear drainage to enhance hydration, but use cautiously with cytotoxic medications [21]
Contact Lens (bandage contact lens, scleral lens)	Provides a protective barrier and retains moisture to promote epithelial healing [2,20,34]
Oral Tetracyclines	Reduces inflammation by inhibiting MMPs, but may affect gut microbiota [5,35,36]
Autologous Serum Drops	Blood-derived drops that supply growth factors and nutrients [37]
Recombinant Human Nerve Growth Factor (rh-NGF) Drops	Cenegermin (a rh-NGF) improves epithelial healing in neurotrophic keratitis-associated PCED [38,39,40]
Insulin Drops	Promotes re-epithelialization and defect closure [41,42]

**Table 2:** Brief descriptions of current surgical managements.

Treatment	Descriptions
Amniotic Membrane Transplant (AMT)	Dressing that promotes epithelialization with anti-inflammatory and anti-scarring properties [24]
Tarsorrhaphy	Partial or full closure of the eyelid to protect and support healing [49]
Conjunctival Flap (CF) Surgery	(Reserved for severe PCEDs) Provides vascular support and reduces inflammation and pain [50]
Limbal Stem Cell Transplantation	Conjunctival–limbal autograft (CLAU) [51] Cultivated limbal epithelial transplantation (CLET) [52] Simple limbal epithelial transplantation (SLET) [53]
Keratoplasty	Replaces damaged corneal tissue, often combined with limbal or neuronal reconstruction [54]
Stromal Puncture	Enhances epithelial adhesion to the basement membrane and prevents recurrent corneal erosions [55]

**Table 3:** Brief descriptions of emerging non-surgical managements.

Treatment	Study	Descriptions
Platelet-Derived Therapeutics	Varies	Platelet-rich plasma [65,66,67] Platelet-rich growth factors [65,68,69] Platelet lysates [70,71,72]
Ocular Bandage Gel	Phase II	Hyaluronic acid crosslinked and stabilized in a bandage gel vehicle [73,74]
Lufeprisen	Phase II/III	Ophthalmic gel that inhibits gap junction hemichannels [75,76,77,78]
0.1% RGN-259	Phase III	Thymosin ß4, an active form of thymosin fraction 5, promotes wound healing [79]
FGLM-NH2 + SSSR	Phase II	FGLM-NH2, a substance *p*-derived peptide, and SSSR, a derivative from the C-domain of insulin-like growth factor-1, promote epithelial regeneration [80,81,82]

**Table 4:** Brief descriptions of emerging surgical managements.

	Treatment	Descriptions
Corneal Neurotization		Restores corneal innervation using nerve grafts to improve sensation and corneal healing [94]
Limbal Cell Reconstruction with Novel Scaffolds	Plywood-like Matrices	Biomimetic scaffold that supports corneal epithelial cell growth with enhanced cell amplification [95]
	Compressed Collagen Gels	Supportive structure for corneal limbal epithelial cell expansion with stronger cell adhesion [96]
	Hyaluronan (HA)	Stimulates corneal epithelial migration and serves as a scaffold for stem cell expansion [97,98]
	Chitosan	A bioresorbable polysaccharide that functions as a scaffold for in situ corneal reconstruction [99,100]
Limbal Cell Reconstruction with Novel Cell Sources	Simple Oral Mucosal Epithelial Transplantation (SOMET)	Autologous graft of oral mucosal epithelial cells for corneal surface restoration [101,102,103,104]
MSC Interventions	Subconjunctival MSC injection	Ongoing phase 2 clinical trial on subconjunctival injections of human bone marrow-derived MSC [105,106,107]
	MSC Transplantation (MSCT)	Mesenchymal stem cells from various sources for ocular surface regeneration [108,109]
	MSC Secretomes	Ongoing phase 2 clinical trials on topical ophthalmic solution containing MSC secreted factors [110,111]

## Data Availability

No new data were created or analyzed in this study.

## Abbreviations

The following abbreviations are used in this manuscript:

PCED	Persistent Corneal Epithelial Defect
LSCD	Limbal Stem Cell Deficiency
MMP	Matrix Metalloproteinase
AMT	Amniotic Membrane Transplant
ASD	Autologous Serum Drops
NK	Neurotrophic Keratitis
TST	Tape Splint Tarsorrhaphy
BCVA	Best-Corrected Visual Acuity
CF	Conjunctival Flap
CLAU	Conjunctival–limbal Autograft
CLET	Cultivated Limbal Epithelial Transplantation
PDGF	Platelet-Derived Growth Factor
PRGF	Platelet Rich in Growth Factors
DCN	Corneal Neuronization
OMEC	Oral Mucosal Epithelial Cells
SOMET	Simple Oral Mucosal Epithelial Transplantation
COMET	Cultivated Oral Mucosal Epithelial Transplantation
MSCT	Mesenchymal Stem Cell Transplantation
CLET	Cultivated Limbal Epithelial Transplantation
BCVA	Best-Corrected Visual Acuity
ETE	End-To-End
STS	Side-To-Side
ETS	End-To-Side
